# Novel method for evaluating the indication for endoscopic papillectomy in patients with ampullary adenocarcinoma

**DOI:** 10.1038/s41598-020-79836-4

**Published:** 2021-01-12

**Authors:** Kenjiro Yamamoto, Takao Itoi, Naoyoshi Nagata, Atsushi Sofuni, Takayoshi Tsuchiya, Kentaro Ishii, Reina Tanaka, Ryosuke Tonozuka, Mitsuyoshi Honjo, Shuntaro Mukai, Yasutsugu Asai, Yukitoshi Matsunami, Hiroshi Yamaguchi, Jun Matsubayashi, Eri Joyama, Yuichi Nagakawa

**Affiliations:** 1 Department of Gastroenterology and Hepatology, Tokyo Medical University, 6-7-1 Nishishinjuku, Shinjuku-ku, Tokyo, 160-0023 Japan; 2 Department of Gastroenterological Endoscopy, Tokyo Medical University, Tokyo, Japan; 3 Department of Anatomic Pathology, Tokyo Medical University, Tokyo, Japan; 4 Department of International Medical Care, Tokyo Medical University, Tokyo, Japan; 5 Third Department of Surgery, Tokyo Medical University, Tokyo, Japan

**Keywords:** Gastrointestinal cancer, Gastrointestinal diseases

## Abstract

This study aimed to determine the clinicopathological features of the subtypes of ampullary carcinoma (AC) to explore the indications for endoscopic papillectomy (EP) in early AC. Fifty-seven patients with AC who underwent curative resection were retrospectively reviewed. The 0/IA stages were significantly more common in the intestinal type (I-type) than in the mixed and pancreatobiliary type (M&PB-type) (90.7% vs 35.7%, *P* < 0.001). Tis/T1a tumors limited to the ampulla [Tis/T1a(ampulla)] were significantly more likely to be I-type than M&PB-type (74.4% vs 14.3%, *P* = 0.002). The tub1 rate was significantly higher in the I-type than in the M&PB-type (81.4% vs 35.7%, *P* = 0.001). In the I-type, the tub1 rate was significantly higher for Tis/T1a(ampulla) than for T1a tumors limited to the sphincter of Oddi (100% vs 42.9%, *P* = 0.004). These observations suggest that I-type AC with tub1 is an indication for EP. The concordance rate of pathological subtypes between endoscopic biopsy and resected specimens was high (κ = 0.8053, *P* < 0.001). Tis/T1a(ampulla) showed no lymphovascular or perineural invasion. An endoscopic imaging finding of early AC with I-type and tub1 on biopsy could be an indication for EP. Identifying the pathological subtype of AC by endoscopic biopsy could be a novel preoperative approach for evaluating the indications for EP.

## Introduction

In recent years, endoscopic papillectomy (EP) has been attempted not only for benign lesions but also for early ampullary carcinoma (AC), defined as a lesion within the sphincter of Oddi on endoscopic ultrasonography (EUS) or intraductal ultrasonography (IDUS)^1,2^. Yamamoto et al. proposed that early AC lesions limited to the ampulla of Vater are an indication for EP because there is no risk of lymphovascular or perineural invasion, lymph node metastasis, or recurrence during long-term follow-up^3^. In contrast, early AC lesions limited to the sphincter of Oddi have a risk of lymphovascular and perineural invasion^3^. However, endoscopic imaging alone cannot determine whether EP is indicated in early AC because it is not possible to determine whether invasion of the sphincter of Oddi is present by EUS or IDUS. Therefore, there is a need for a preoperative method that can distinguish between lesions limited to the ampulla of Vater and those limited to the sphincter of Oddi.

AC can now be classified pathologically as intestinal type (I-type), mixed type (M-type), or pancreatobiliary type (PB-type) according to the epithelium from which the adenocarcinoma originates^4–10^. Several studies have suggested that the M-type and PB-type of AC have a less favorable prognosis than the I-type^11,12^. However, it is unclear whether there are any differences in clinicopathological features between the subtypes in early AC. We hypothesized that the pathological subtype could differentiate between a lesion limited to the ampulla of Vater and a lesion limited to the sphincter of Oddi. The aim of this study was to determine the clinicopathological features of the subtypes of AC and to explore a preoperative method that can distinguish between lesions limited to the ampulla of Vater and those limited to the sphincter of Oddi, that is, the indications for EP in early AC.

## Results

The clinicopathological characteristics of the patients are shown in Table 1. The pathological subtype was I-type in 43 patients (75.4%), M-type in 8 (14%), and PB-type in 6 (10.5%). In early AC, the overall stage was 0 in 8 patients (14%) and IA in 36 patients (63.2%); T1a(ampulla) was the most common primary tumor (28 patients, 49.1%) followed by Tis (8 patients; 14%) and T1a(Oddi) (8 patients; 14%). The most common pathological grade was well-differentiated tubular adenocarcinoma (tub1; 40 patients, 70.2%), followed by moderately differentiated tubular adenocarcinoma (tub2; 13 patients, 22.8%), and poorly differentiated adenocarcinoma (por; 4 patients, 7%).

**Table 1:** Patient demographics and clinicopathological characteristics.

Patients, n	57
Age, years, median (range)	71 (41–85)
Sex, male/female	36/21
Tumor size, mm, mean (range)	17.8 (5–47)
**Type of treatment, n (%)**	
Endoscopic papillectomy	38 (66.7)
Surgical resection	19 (33.3)
**Pathological subtype**	
Intestinal	43 (75.4)
Mixed	8 (14.0)
Pancreatobiliary	6 (10.5)
**Overall stage, n (%)**	
0	8 (14.0)
IA	36 (63.2)
IB	4 (7.0)
IIA	2 (3.5)
IIIB	0
IIIA	7 (12.3)
**T stage of primary tumor, n (%)**	
Tis	8 (14.0)
T1a (ampulla)	28 (49.1)
T1a (Oddi)	8 (14.0)
T1b	0
T2	7 (12.3)
T3a	5 (8.8)
T3b	1 (1.8)
**Pathological grade, n (%)**	
Well differentiated	40 (70.2)
Moderately differentiated	13 (22.8)
Poorly differentiated	4 (7)
**Invasion**	
Lymphatic, n (%)	15 (26.3)
Venous, n (%)	10 (17.5)
Perineural, n (%)	6 (10.5)
Lymph node metastasis, n (%)	7 (12.3)

Overall disease stage and T stage of the primary tumor are based on the World Health Organization classification (0, TisN0M0; IA, T1aN0M0; IB, T1b/T2N0M0; IIA, T3aN0M0; IIB, T3bN0M0; IIIA, T1a/T1b/T2/T3N1M0). Tis, carcinoma in situ; T1a(ampulla), tumor limited to the ampulla of Vater; T1a(Oddi), tumor limited to the sphincter of Oddi; T1b, tumor extends beyond the sphincter of Oddi and/or into the duodenal submucosa; T2, tumor invades the muscularis propria of the duodenum; T3a, tumor invades 0.5 cm or less into the pancreas; T3b, tumor invades more than 0.5 cm into the pancreas or extends into the peripancreatic tissue or duodenal serosa but without involvement of the celiac axis or the superior mesenteric artery.

### Pathological analysis

**Figure 1:**
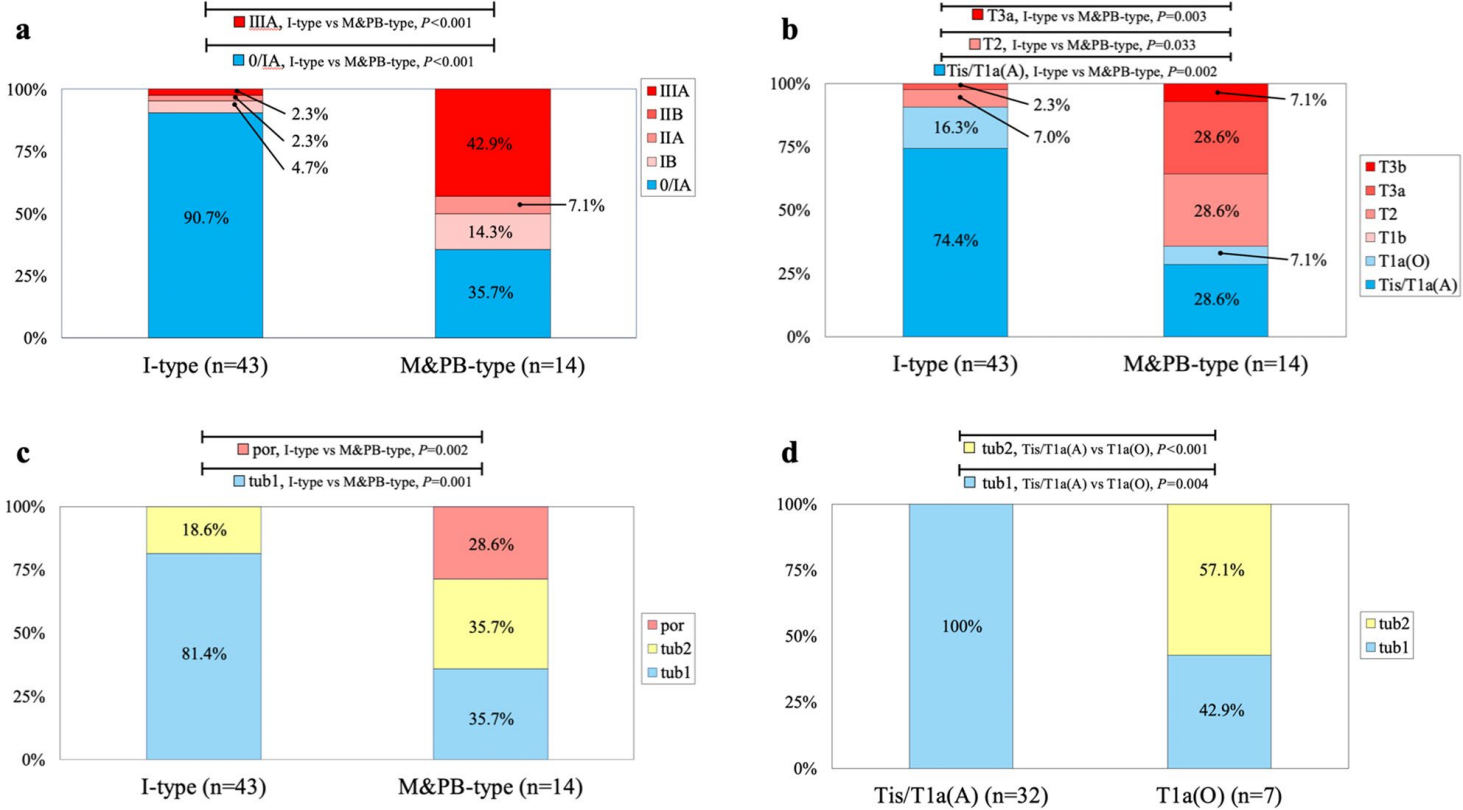
Comparison of overall stage, T stage of the primary tumor, and pathological grade between the intestinal-type and mixed/pancreatobiliary-type. (**a**) Overall stage. (0, TisN0M0; IA, T1aN0M0; IB, T1b/T2N0M0; IIA, T3aN0M0; IIB, T3bN0M0; IIIA, T1a/T1b/T2/T3N1M0). There were statistically significant differences in IA (*P* = 0.014) and IIIA (*P* < 0.001) between the intestinal and mixed/pancreatobiliary types. There was no significant difference in 0, IB, IIA, or IIB between the intestinal and mixed/pancreatobiliary types. (**b**) T stage of the primary tumor. Tis, carcinoma in situ; T1a(A), tumor limited to ampulla of Vater; T1a(O), tumor limited to sphincter of Oddi; T2, tumor invades the muscularis propria of the duodenum; T3a, tumor invades 0.5 cm or less into the pancreas. There were significant differences in Tis and T1a(A) (*P* = 0.002), T2 (*P* = 0.033), and T3a (*P* = 0.003) between the intestinal and mixed/pancreatobiliary types. There was no significant difference in T1a(O), T1b, or T3b between the intestinal and mixed/pancreatobiliary types. (**c**) Pathological grade. tub1, well-differentiated tubular adenocarcinoma; tub2, moderately differentiated tubular adenocarcinoma; por, poorly differentiated adenocarcinoma. There were significant differences in tub1 (*P* = 0.001) and por (*P* < 0.002) between the intestinal and mixed/pancreatobiliary types. There was no significant difference in tub2 between the intestinal and mixed/pancreatobiliary types. (**d**) Pathological grade of intestinal-type early AC. tub1, well-differentiated tubular adenocarcinoma; tub2, moderately differentiated tubular adenocarcinoma; Tis, carcinoma in situ; T1a(A), tumor limited to ampulla of Vater; T1a(O), tumor limited to sphincter of Oddi. There were significant differences in tub1 (100% vs 42.9%, *P* = 0.004) and tub2 (0% vs 57.1%, *P* < 0.001) between the Tis/T1a(A) and T1a(O) groups.

The pathological characteristics are compared between the I-type and the mixed and pancreatobiliary type (M&PB-type) in Fig. 1a–d. The pathological characteristics of each subtype are shown in Supplementary Fig. S1a–d. The PB-type tumors were smaller than the I-type and M-type tumors. There was a significant difference in size between the M-type and PB-type tumors (*P* = 0.03; Supplementary Fig. S1a).

Almost all I-type tumors had an overall stage of 0/IA (i.e., early AC) whereas more than half of the M&PB-type tumors were higher than stage IB (i.e., advanced AC). The 0/IA stage was significantly more common in the I-type than in the M&PB-type (90.7% vs 35.7%, *P* < 0.001; Fig. 1a, Supplementary Fig. S1b). I-type primary tumors had high rates of early AC whereas the M&PB-types had high rates of advanced AC. Tis/T1a(ampulla) was significantly more likely in the I-type than in the M&PB-type (74.4% vs 14.3%, *P* = 0.002; Fig. 1b, Supplementary Fig. S1c). A pathological grade of tub1 was more common in the I-type whereas tub2 and por were more common in the M&PB-type. The tub1 rate was significantly higher in the I-type than in the M&PB-type (81.4% vs 35.7%, *P* = 0.001; Fig. 1c, Supplementary Fig. S1d). In the I-type, the tub1 rate was significantly higher in Tis/T1a(ampulla) tumors than in T1a(Oddi) tumors (100% vs 42.9%, *P* = 0.004; Fig. 1d).

**Figure 2:**
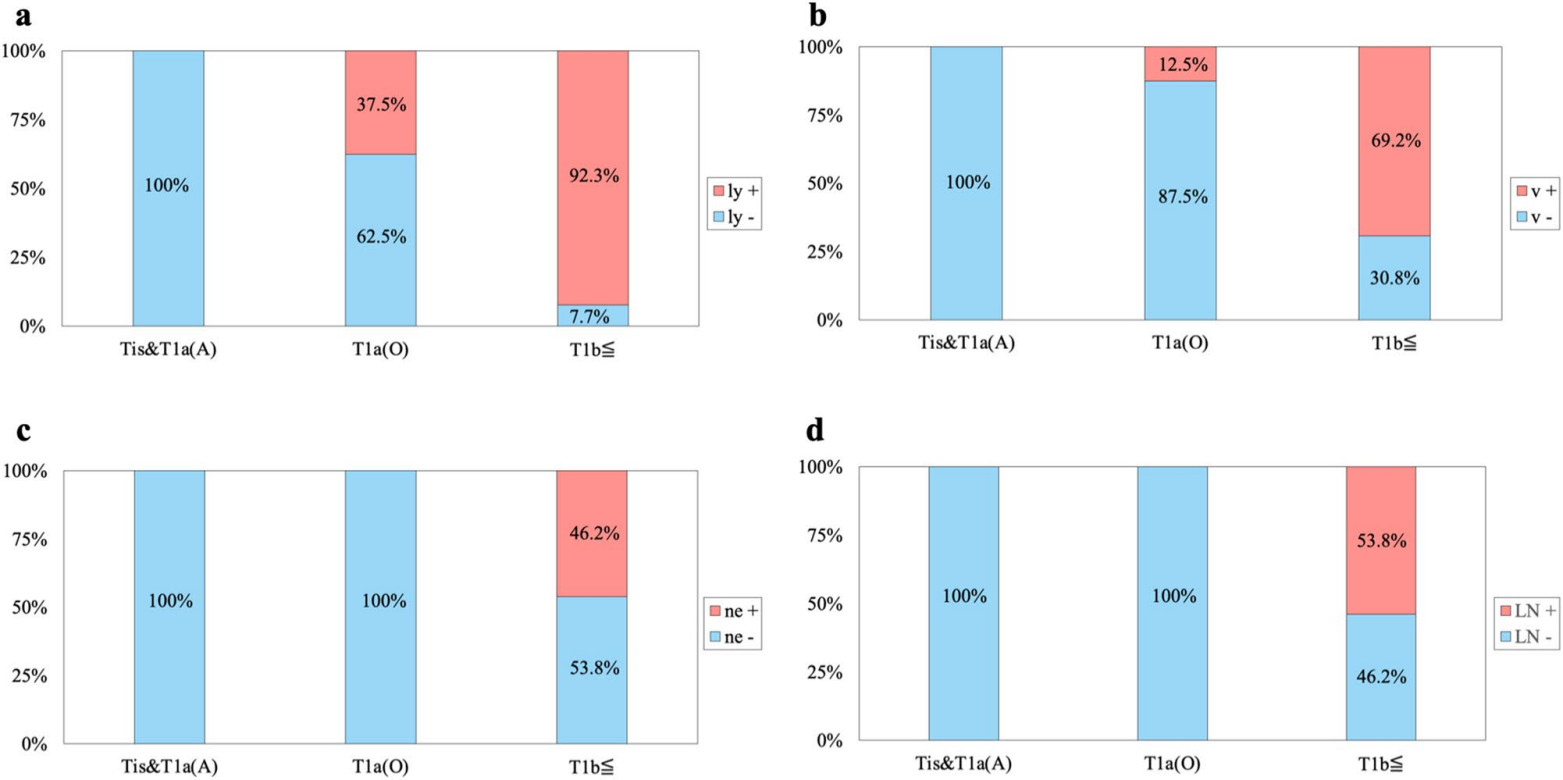
Pathological tumor characteristics of tumors with lymphatic, vascular, or perineural invasion and lymph node metastasis. (**a**) Lymphatic invasion. (**b**) Vascular invasion. (**c**) Perineural invasion. (**d**) Lymph node metastasis. In a–d, the number of intestinal-type Tis/T1a(A), T1a(O), and ≥ T1b lesions were 36, 8, and 13, respectively.

None of the Tis/T1a(ampulla) samples showed lymphovascular or perineural invasion and none of the Tis/T1a(ampulla) or T1a(Oddi) samples had lymph node metastases (Fig. 2).

### Concordance between endoscopic biopsy and resected specimens for tumor diagnosis

There was high diagnostic concordance between the specimens obtained by endoscopic biopsy and specimens resected by endoscopic papillectomy or surgery in terms of pathological subtype (κ = 0.805; 95% confidence interval [CI], 0.55–1.06, *P* < 0.001) and pathological grade (κ = 0.645; 95% CI 0.30–0.99, *P* = 0.001; Table 2, Supplementary Table S2).

**Table 2:** Diagnostic concordance of pathological subtype and grade between endoscopic biopsy specimens and resected specimens (n = 22).

Pathological subtype		
Endoscopic biopsy specimens	Resected specimens	
	I-type (n = 15)	M&PB-type (n = 7)
I-type (n = 13)	13	0
M&PB-type (n = 9)	2	7
κ = 0.805 (95% CI, 0.55–1.06), *P* < 0.001		
CI, confidence interval, I-type, intestinal type; M&PB-type, mixed and pancreatobiliary type		

Pathological grade		
Endoscopic biopsy specimens	Resected specimens	
	tub1 (n = 15)	tub2 or por (n = 7)
tub1* (n = 18)	15	3
tub2 or por (n = 4)	0	4
κ = 0.645 (95% CI, 0.30–0.99), *P* = 0.001		
CI, confidence interval; tub1, well differentiated; tub2, moderately differentiated; por, poorly differentiated; tub1* includes high-grade adenoma		

## Discussion

This study had four important findings. First, Tis/T1a(ampulla) tumors were significantly more likely to be the I-type than the M&PB-type. Second, there was a significantly higher rate of tub1 in the I-type than in the M&PB-types. Third, in the I-type, the tub1 rate was significantly higher in Tis/T1a(ampulla) tumors than in T1a(Oddi) tumors. These observations suggest that I-type AC with tub1 could be an indication for EP. Finally, there was a high concordance rate for pathological subtype between endoscopic biopsy and resected specimens, suggesting that biopsy is adequate for pathological evaluation. To our knowledge, this is the first study to investigate the clinicopathological features of the pathological subtypes of early AC. Our results suggest that endoscopic imaging with biopsy alone could be useful in determining whether EP is indicated.

Kimura et al. initially reported that AC could be classified as I-type or PB-type based on the epithelium from which the adenocarcinoma originates^7^. Other reports have emphasized the importance of differentiating histologically between these pathological subtypes because of their different prognoses^9,11–16^. Okano et al. demonstrated that overall survival was significantly lower in patients with the PB-type than in those with the I-type because the PB-type is more aggressive in terms of lymphatic, vascular, perineural, and pancreatic invasion^11^. Furthermore, Asano et al.^12^ demonstrated that the pathological characteristics of the mixed type were similar to those of the PB-type and that prognosis was poorer for the M-type than for the I-type.

In our study, 75.4% of ACs were the I-type, 14% were the M-type, and 10.5% were the PB-type whereas the respective proportions have previously been reported as 27–57%, 6.9–8.7%, and 36–60%^5,9,10,16–19^. The higher proportion of I-type ACs in our study may reflect the fact that we included many lesions that were amenable to EP and did not target unresectable lesions with distant metastases, a high proportion of which are likely to be M-type or PB-type ACs with a less favorable prognosis. Detailed analyses of the pathological findings according to AC subtype (Fig. 1) highlighted the following important issues when determining the indication for EP in early AC: (1) I-type tumors have less invasive features with higher rates of Tis/T1a(ampulla) compared with M&PB-type tumors; (2) I-type tumors have higher rates of tub1 than M&PB-type tumors; and (3), in the I-type, the tub1 rate is significantly higher for Tis/T1a(ampulla) lesions than for T1a(Oddi) lesions. These observations suggest that I-type early AC with tub1 is indicated for EP.

We also analyzed the pathological subtypes of AC using MUC1 and MUC2 (Supplementary Table S3). Chu et al.^20^ demonstrated expression of MUC2 (intestinal-type mucin) in ampullary tumors with an intestinal phenotype and MUC1 (pancreatic-type mucin) in those with a pancreaticobiliary phenotype. Similarly, we found that the I-type was negative for MUC1 and positive for MUC2 whereas the PB-type was positive for MUC1 and negative for MUC2. Moreover, the mixed type was positive for both MUC1 and MUC2.

The rate of false-negative diagnosis of ampullary tumors on preprocedural endoscopic biopsy is reported to be high^21–23^. Indeed, although the architectural features of the pathological subtype were consistent regardless of the depth of the lesion, the pathological grade differed occasionally depending on the depth of the lesion (Supplementary Fig. S4). However, we observed high diagnostic concordance of pathological subtype (κ = 0.805; 95% CI, 0.55–1.06, *P* < 0.001) and pathological grade (κ = 0.645; 95% CI, 0.30–0.99, *P* = 0.001) between endoscopic biopsy specimens and resected specimens (Table 2, Supplementary Table S2). These findings indicate that analysis of a biopsy specimen is sufficiently reliable for determination of the pathological features of the tumor.

Finally, we propose the following strategy for treatment of early AC lesions based on pathological subtype (Supplementary Fig. S5). I-type ACs with tub1 have a high likelihood of being Tis/T1a(ampulla). Therefore, these lesions are an indication for EP. However, I-type ACs without tub1 are less likely to be Tis/T1a(ampulla) and require surgery. M-type ACs have an intermediate prognosis and surgery is preferable to EP. Although 4 (50%) of 8 M-type ACs in this study were Tis/T1a(ampulla), this is not sufficient to assume an indication for EP because of our small sample size (Supplementary Fig. S1c). PB-type ACs, which have the least favorable prognosis, are a contraindication for EP and require surgery (Supplementary Figs. S1b,c,d).

The main limitations of this study were the small number of cases, the lack of data regarding unresectable T4 AC lesions, and the retrospective single-center design. Therefore, a degree of selection bias cannot be excluded. Furthermore, this study evaluated pathological assessment based on curative resected specimens but did not examine the outcomes of EP itself. However, the main goal of this study was to narrow down the early AC lesions for which the indications of EP can be expanded. Therefore, the pathological subtype is likely to be one of the important factors for narrowing down these lesions.

In conclusion, identifying the pathological subtype of AC during endoscopic biopsy is a novel preoperative approach to determining the indication for EP. An endoscopic imaging finding of early AC of I-type with tub1 on biopsy could be an indication for EP. Future large-scale prospective studies that include the long-term outcome are needed to determine the exact frequency of each subtype and confirm whether identifying the clinicopathological features of the subtypes of AC is a suitable a preoperative method to determine the indications for EP in early AC.

## Methods

### Patients

Data for 57 patients who underwent curative resection of AC (EP, n = 38; pancreatoduodenectomy, n = 19) at Tokyo Medical University Hospital between April 2005 and December 2017 were reviewed. Two of the 19 pancreatoduodenectomy cases underwent additional surgery after complete resection could not by achieved by EP. The clinical data were analyzed retrospectively and paraffin blocks of specimens from the 57 patients were prospectively prepared for pathological and immunohistochemical (IHC) analyses to determine the subtype. Preoperative evaluation of lymph node metastases was based on the presence or absence of enlarged regional lymph nodes on diagnostic imaging (computed tomography, magnetic resonance imaging, or EUS). The study was approved by the institutional review board of Tokyo Medical University (approval number, 4081) and performed in accordance with the relevant guidelines and regulations (Declaration of Helsinki). Informed consent was obtained from all study participants.

### Pathological analysis

Slides stained with hematoxylin–eosin were analyzed to determine tumor size, pathological grade, depth of invasion, duodenal or pancreatic invasion, lymphovascular and perineural invasion, and lymph node metastasis. Overall stage and T stage of the primary tumor were classified according to the TNM system (Supplementary Figs. S6, S7). For convenience, we subdivided T1a lesions based on the World Health Organization (WHO) classification into those that were T1a(ampulla) and those that were T1a(Oddi). The pathological subtype of each tumor was determined based on the WHO classification guidelines by a single pathologist (HY) who was blinded to the clinical findings. Most I-type and PB-type lesions could be diagnosed pathologically by their architectural features in hematoxylin–eosin-stained samples; however, it was occasionally difficult to diagnose M-type lesions because this type has architectural features of both I-type and PB-type lesions. Therefore, we defined the pathological subtypes of AC using MUC1 and MUC2 because MUC2 is found in ampullary tumors with an intestinal phenotype and MUC1 is found in those with a pancreaticobiliary phenotype.

### Subtype definitions

**Figure 3:**
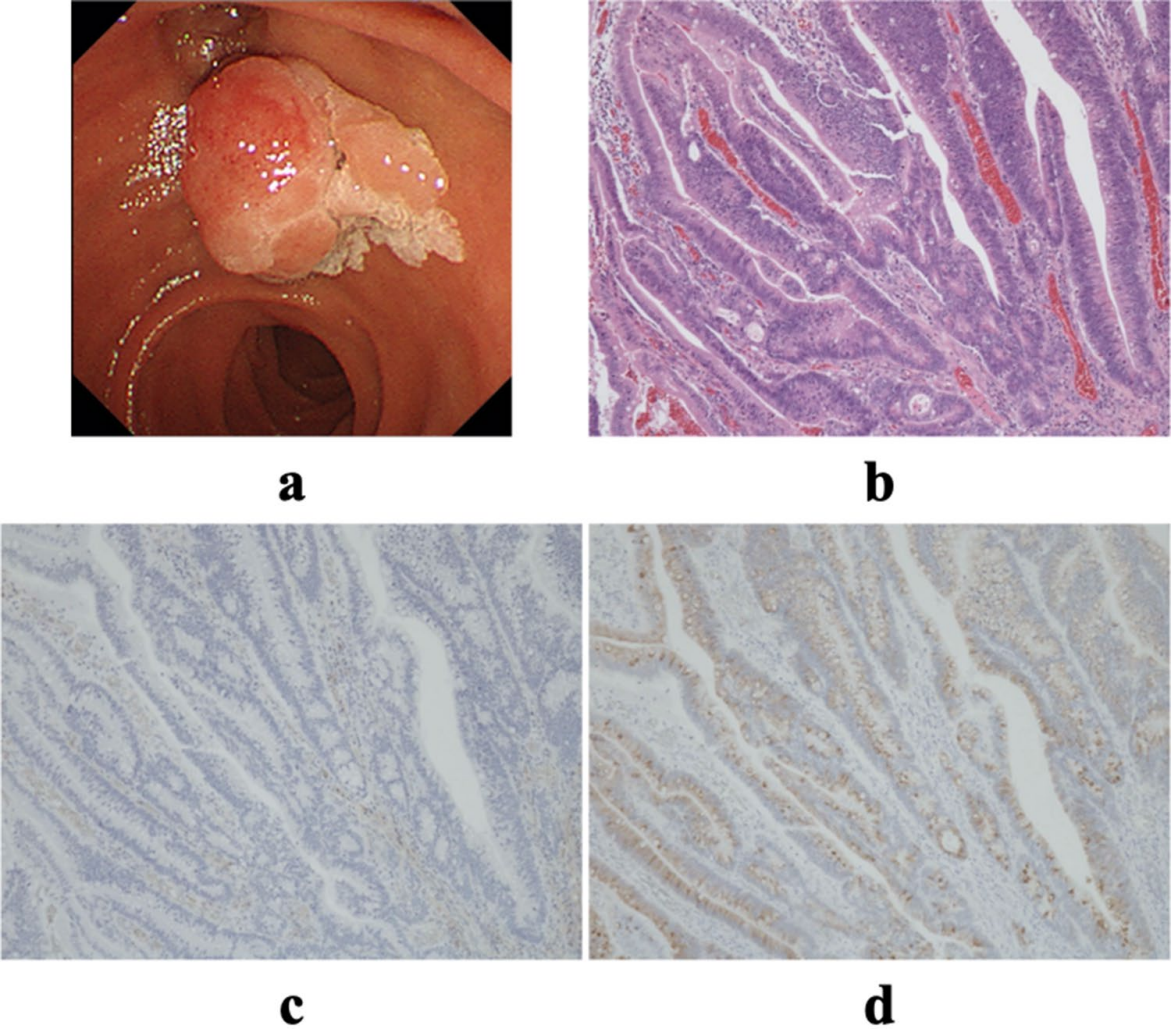
Characteristics of intestinal-type tumors. (**a**) Endoscopic image. (**b**) Hematoxylin–eosin staining of the tumor (× 40). (**c**) MUC1 expression in the tumor (× 40); (**d**) MUC2 expression in the tumor (× 40). MUC1, pancreatic-type mucin; MUC2, intestinal-type mucin.

**Figure 4:**
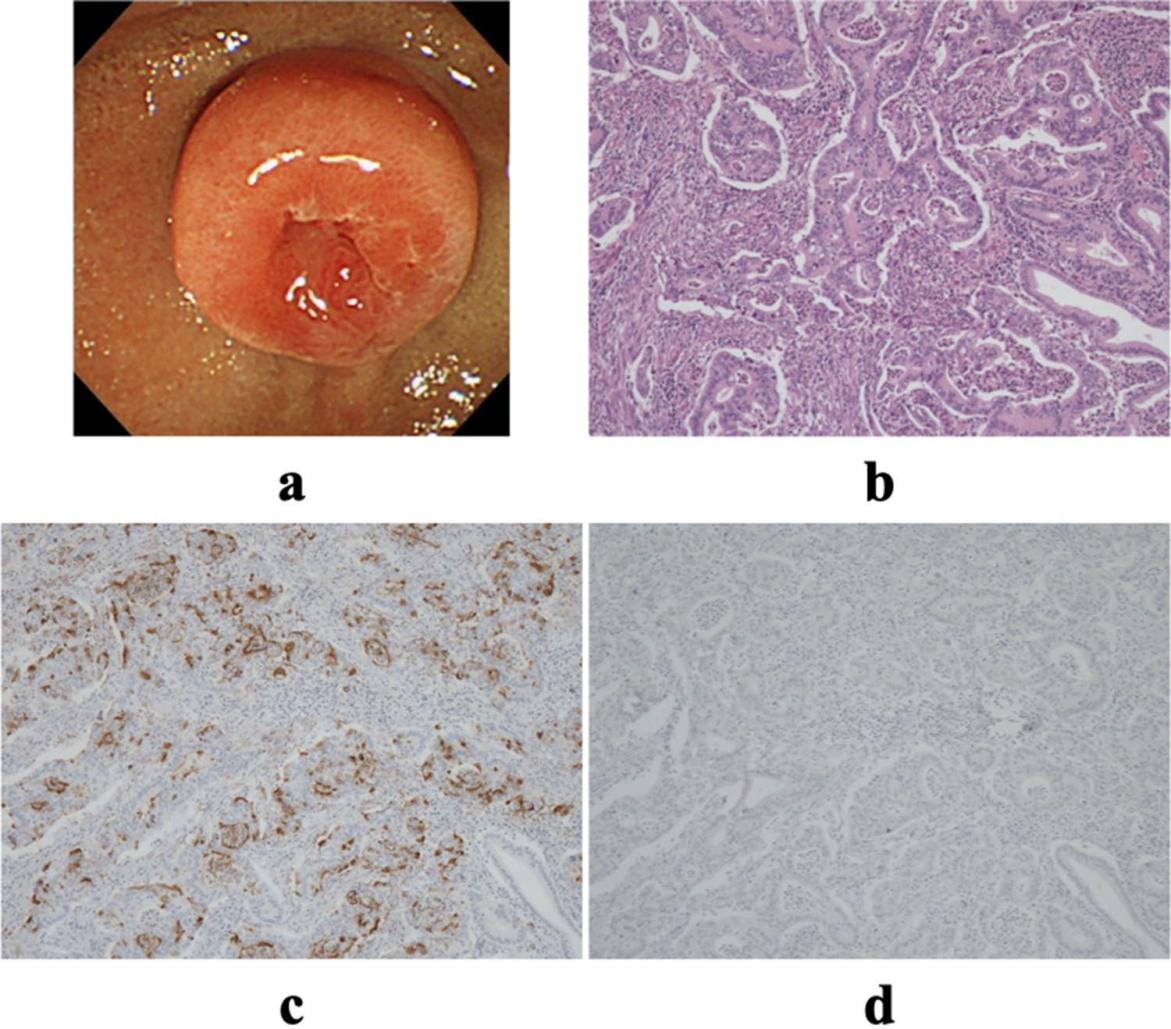
Characteristics of pancreatobiliary-type tumors. (**a**) Endoscopic image. (**b**) Hematoxylin–eosin staining of the tumor (× 40). (**c**) MUC1 expression in the tumor (× 40). (**d**) MUC2 expression in the tumor (× 40). MUC1, pancreatic-type mucin; MUC2, intestinal-type mucin.

**Figure 5:**
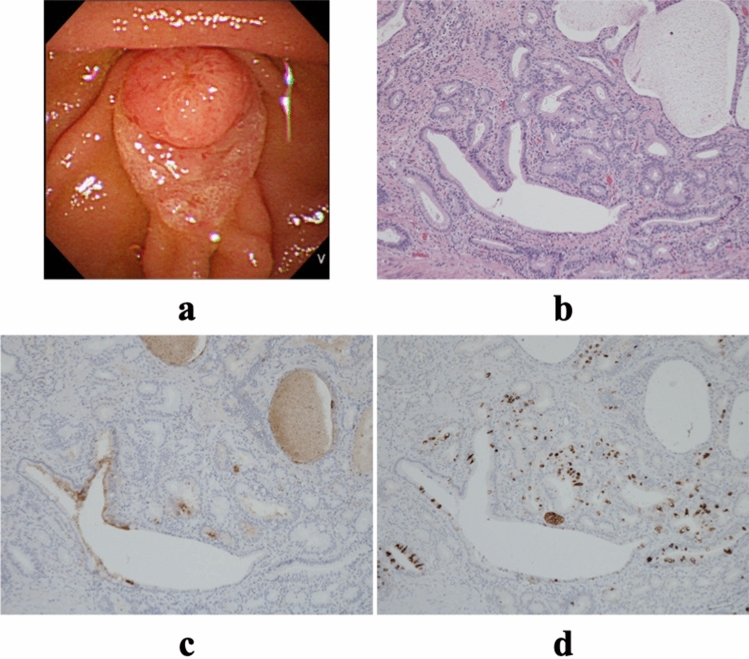
Characteristics of mixed-type tumors. (**a**) Endoscopic image. (**b**) Hematoxylin–eosin staining of the tumor (× 40). (**c**) MUC1 expression in the tumor (× 40). (**d**) MUC2 expression in the tumor (× 40). MUC1, pancreatic-type mucin; MUC2, intestinal-type mucin.

The endoscopic findings for each subtype of AC are shown in Figs. 3a, 4a, and 5a.

#### I-type

These gland (tubule)-forming adenocarcinomas are morphologically similar to colon adenocarcinoma. The glands are lined by tall columnar cells with elongated, pseudostratified nuclei. Scattered goblet, Paneth, and neuroendocrine cells may be present^24–26^. Occasionally, I-type adenocarcinomas contain dysplastic glandular epithelium with mild architectural complexity and mild nuclear atypia (Fig. 3b).

#### PB-type

This type of adenocarcinoma is composed of relatively small glandular units that are widely separated by demoplastic stroma similar to pancreatic ductal, gallbladder, and extrahepatic biliary tract adenocarcinoma. The epithelium is usually a single layer of cuboidal to low columnar cells without substantial nuclear pseudostratification but with more pleiomorphism than the I-type^24,27,28^ (Fig. 4b).

#### M-type

Many carcinomas in the ampulla have ambiguous features that are difficult to classify definitively as the I-type or PB-type. These ambiguous cases account for more than a third of all AC cases^24,27,28^ and should be classified as mixed type. If the specimen contained both intestinal and pancreatobiliary regions, the sample was categorized as the subtype that comprised the larger area within the sample. However, if intestinal and pancreatobiliary regions each comprised more than 20% of the sample area, the AC was classified as M-type (Fig. 5b).

### Immunohistochemical analysis

All samples were stained with both anti-MUC1 (Ma695, 1:200 dilution) and anti-MUC2 (Ccp58, 1:100 dilution). Both antibodies were obtained from Leica Microsystems (Wetzlar, Germany). IHC staining was performed using a Leica BOND III automatic immunostainer (Leica Microsystems) after incubation of the sample in a decloaking chamber for antigen activation. All the stained samples were analyzed by the same pathologist (HY). The samples were scored according to the percentage of tumor cells that were stained and were arbitrarily defined as being IHC-positive if more than 30% of tumor cells were positively stained^29^ (Figs. 3c, d, 4c, d, 5c, d).

### Statistical analysis

Patient characteristics are summarized as descriptive data. The pathological characteristics were compared among the I-type, M-type, and PB-type of AC. The Kruskal–Wallis test was used to compare quantitative variables among the three groups and the chi-square test to compare qualitative variables among three groups and between two groups. The test–retest reliability of the concordance between diagnoses made by endoscopic biopsy and those made from surgically resected specimens was analyzed using the kappa statistic (> 0.80, excellent agreement; > 0.60–0.80, good; 0.40–0.60, moderate; > 0.20–0.40, fair; and ≤ 0.20, poor)^30^ with 95% CIs. All statistical analyses were performed using SPSS version 13.0 software (SPSS Inc., Chicago, IL, USA). A *P*-value of < 0.05 was considered to indicate a statistically significant difference between two groups or three groups.

## Supplementary information

Supplementary Information.
