# Supplementary material for: Novel method for evaluating the indication for endoscopic papillectomy in patients with ampullary adenocarcinoma
Source: Sci Rep. 2021 Jan 12;11:600. doi: 10.1038/s41598-020-79836-4 (PMC7804087; doi:10.1038/s41598-020-79836-4)
Supplement: Supplementary file 1 — Supplementary Information. [file 41598_2020_79836_MOESM1_ESM.pdf]

Novel method for evaluating the indication for endoscopic papillectomy in patients with ampullary adenocarcinoma

Kenjiro Yamamoto[1], Takao Itoi[1*], Naoyoshi Nagata[2], Atsushi Sofuni[1], Takayoshi Tsuchiya[1], Kentaro Ishii[1], Reina Tanaka[1], Ryosuke Tonozuka[1], Mitsuyoshi Honjo[1], Shuntaro Mukai[1], Yasutsugu Asai[1], Yukitoshi Matsunami[1], Hiroshi Yamaguchi[3], Jun Matsubayashi[3], Eri Joyama[4], Yuichi Nagakawa[5]

[1] *Department of Gastroenterology and Hepatology, Tokyo Medical University, Tokyo, Japan*
[2] *Department of Gastroenterological Endoscopy, Tokyo Medical University, Tokyo, Japan*
[3] *Department of Anatomic Pathology, Tokyo Medical University, Tokyo, Japan*
[4] *Department of International Medical Care, Tokyo Medical University, Tokyo, Japan*
[5] *Third Department of Surgery, Tokyo Medical University, Tokyo, Japan*

Corresponding author: Takao Itoi, MD, PhD, FACG, FASGE
Department of Gastroenterology and Hepatology
Tokyo Medical University
6-7-1 Nishishinjuku, Shinjuku-ku, Tokyo 160-0023, Japan
Tel.: +81-3-3342-6111; Fax: +81-3-5381-6654
E-mail: itoi@tokyo-med.ac.jp

Supplementary Figure S1. Pathological tumor characteristics, diameter, overall stage, T stage of the primary tumor, and pathological grade according to subtype

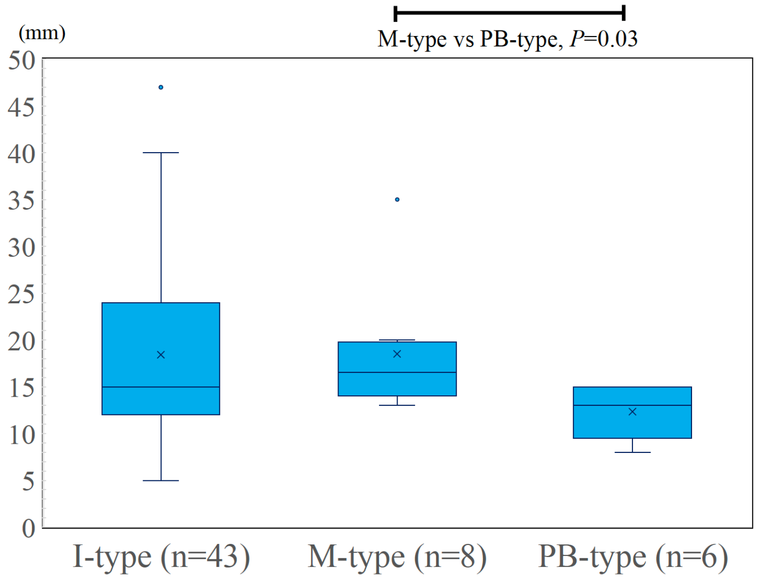

(**a**) Tumor diameter.

Median diameters of the I-type, M-type, and PB-type lesions were 15 mm, 16.5 mm, and 13 mm, respectively. There was no statistically significant difference (*P*=0.1665) among the three groups (Kruskal-Wallis test). Significant differences were noted between the M- and PB-types (*P*=0.03) but not between the I- and M-types or between the I- and PB-types. I-type, intestinal type; M-type, mixed type; PB-type, pancreatobiliary type.

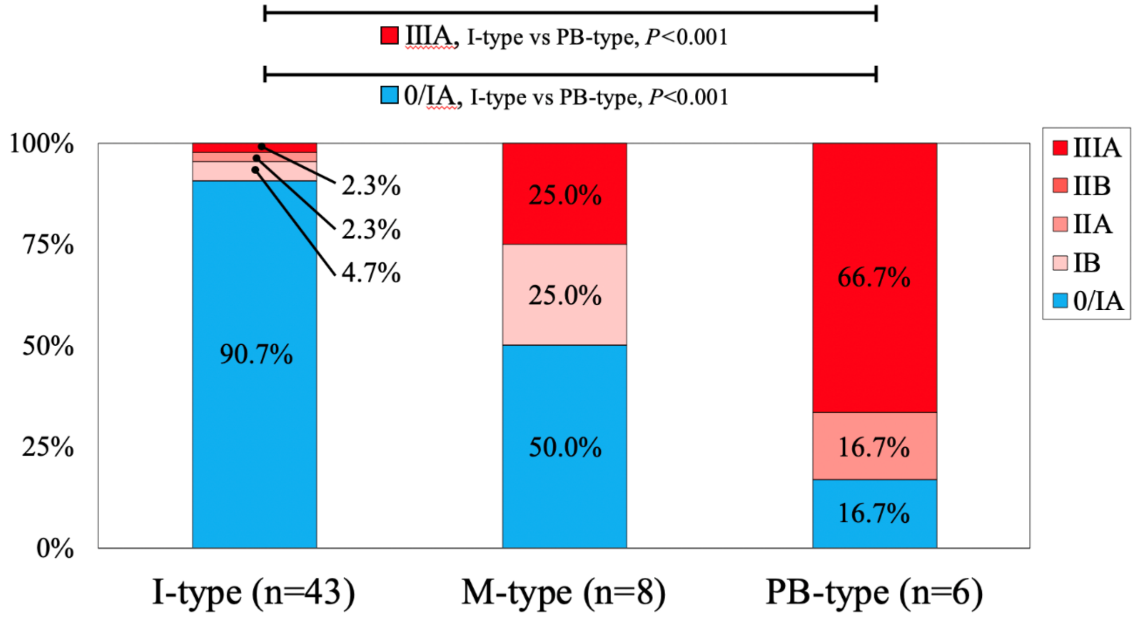

(**b**) Overall stage (0, TisN0M0; IA, T1aN0M0; IB, T1b/T2N0M0; IIA, T3aN0M0; IIB, T3bN0M0; IIIA, T1a/T1b/T2/T3N1M0).

There were statistically significant differences in IA (*P*=0.022) and IIIA (*P*<0.001) among the three groups. There were also significant differences in IA (*P*=0.015) and IIIA (*P*<0.001) between the I- and PB-types. There was no statistically significant difference in 0 between the I- and M-types, the I- and PB-types, or the M- and PB-types, in IA between the I- and M-types or the M- and PB-types, in IB between the I- and M-types, I- and PB-types, or M- and PB-types, in IIA between the I- and M-types, I- and PB-types, or M- and PB-types, in IIB between the I- and M-types, I- and PB-types, or M- and PB-types, or in IIIA between the I- and M-types or M- and PB-types. I-type, intestinal type; M-type, mixed type; PB-type, pancreatobiliary type.

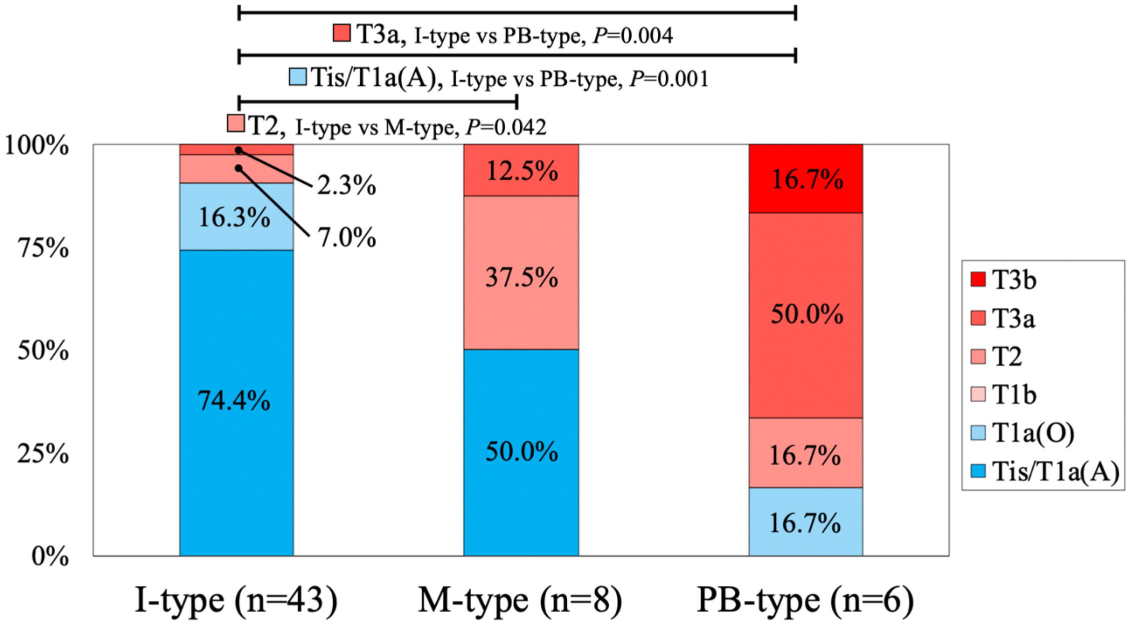

(**c**) T stage of the primary tumor.

There were statistical significances in Tis and T1a(A) (P=0.001) and T3a (P=0.001) among the three groups. There were significant differences in Tis/T1a(A) between the I- and PB-types (P=0.001), in T2 between the I- and M-types (P=0.042), and in T3a between the I- and PB-types (P=0.004). There was no statistically significant difference in Tis/T1a(A) between the I- and M-types or the M- and PB-types, in T1a(O) between the I- and M-types, the I- and PB-types, or the M- and PB-types, or in T1b between the I- and M-types, the I- and PB-types, or the M- and PB-types, in T2 between the I- and PB-types, or M- and PB-types, in T3a between the I- and M-types or the M- and PB-types, or in T3b between the I- and M-types, the I- and PB-types, or the M- and PB-types. Tis, carcinoma in situ; T1a(A), tumor limited to ampulla of Vater; T1a(O), tumor limited to sphincter of Oddi; T2, tumor invading the muscularis propria of the duodenum; T3a, tumor invading 0.5 cm or less into the pancreas; I-type, intestinal type; M-type, mixed type; PB-type, pancreatobiliary type.

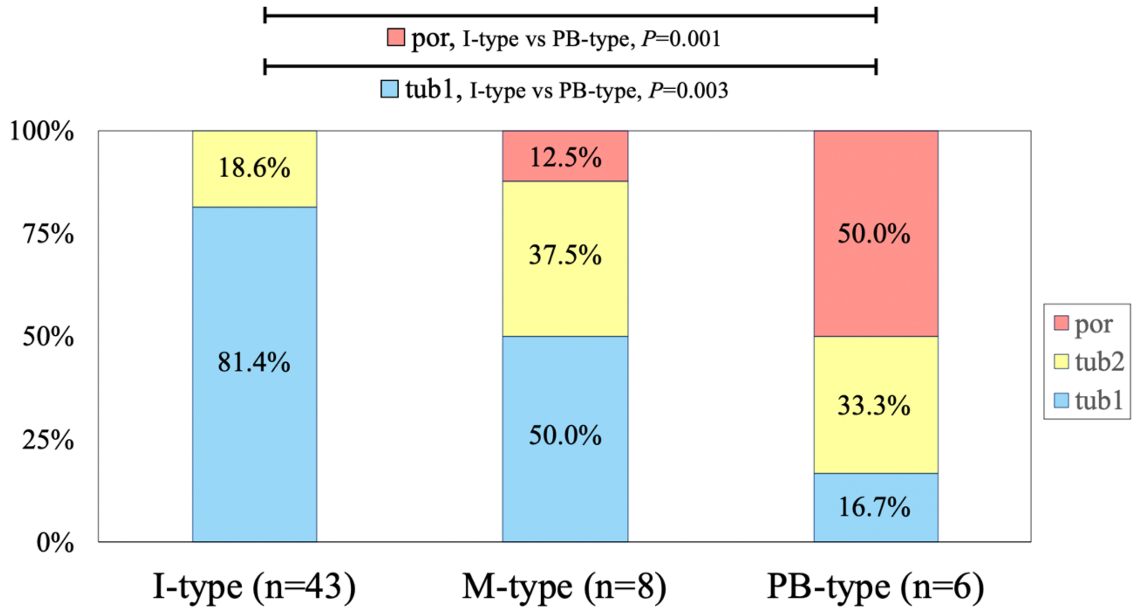

(**d**) Pathological grade.

There were statistically significant differences in tub1 (P=0.002) and por (P<0.001) status among the three groups. There were significant differences in tub1 (P=0.003) and in por (P=0.001) between the I- and PB-types. There was no statistically significant difference in tub1 between the I- and M-types or the M- and PB-types, in tub2 between the I- and M-types, in tub2 between the I- and PB-types, in tub2 between the M- and PB-types, or in por between the I- and M-types and M- and PB-types. tub1, well differentiated tubular adenocarcinoma; tub2, moderately differentiated tubular adenocarcinoma; por, poorly differentiated adenocarcinoma; I-type, intestinal type; M-type, mixed type; PB-type, pancreatobiliary type.

Supplementary Table S2. Concordance for tumor diagnosis between endoscopic biopsy and resected specimens (n=22)

Endoscopic biopsy	Resected specimens		
	I-type (n=13)	M type (n=4)	P -type (n=3)
I-type (n=13)	13	0	0
M-type (n=5)	2	3	0
PB-type (n=4)	0	1	3
κ=0.815 (95% CI, 0.62-1.01), *P*<0.001			

I-type, intestinal type; M-type, mixed type; PB-type, pancreatobiliary type

Supplementary Table S3. Immunohistochemical analyses of pathological subtypes

	**I-type** n=43	**M-type** n=8	**PB-type** n=6
**MUC1**			
Positive, n (%)	3 (7.0)	8 (100)	6 (100)
Negative, n (%)	40 (93.0)	0 (0)	0 (0)
**MUC2**			
Positive, n (%)	37 (86.0)	8 (100)	0 (0)
Negative, n (%)	6 (14.0)	0 (0)	6 (100)

I-type, intestinal type; M-type, mixed type; PB-type, pancreatobiliary type

Supplementary Figure S4. Pathological grade and subtype of tumor according to depth of lesion

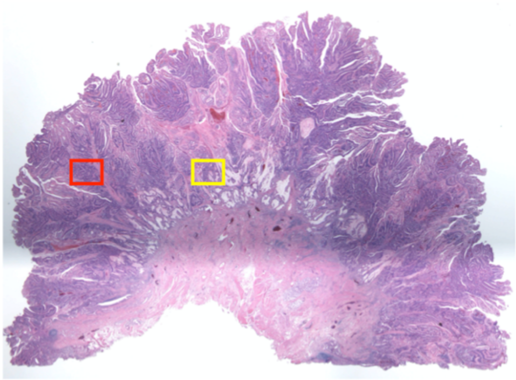

a

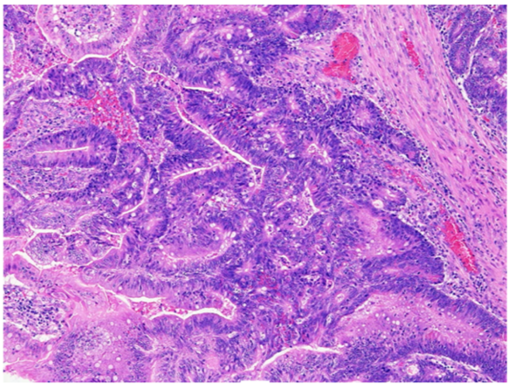

b

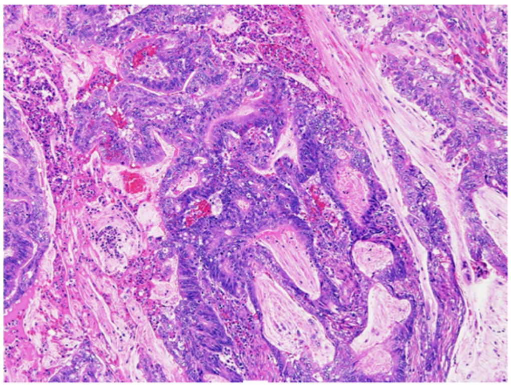

c

(**a**) Intestinal-type ampullary carcinoma. (**b**) Shallow layer of the lesion. (**c**) Deep layer of the lesion. The pathological grade occasionally differs depending on the depth of the lesion (the shallow layer is well differentiated [red square in **a**, **b**] whereas the deep layer is moderately differentiated [yellow square in **a**, **c**]) and the pathological subtypes are distributed consistently.

Supplementary Figure S5. Proposed strategy for treatment of early ampullary carcinoma based on pathological subtype

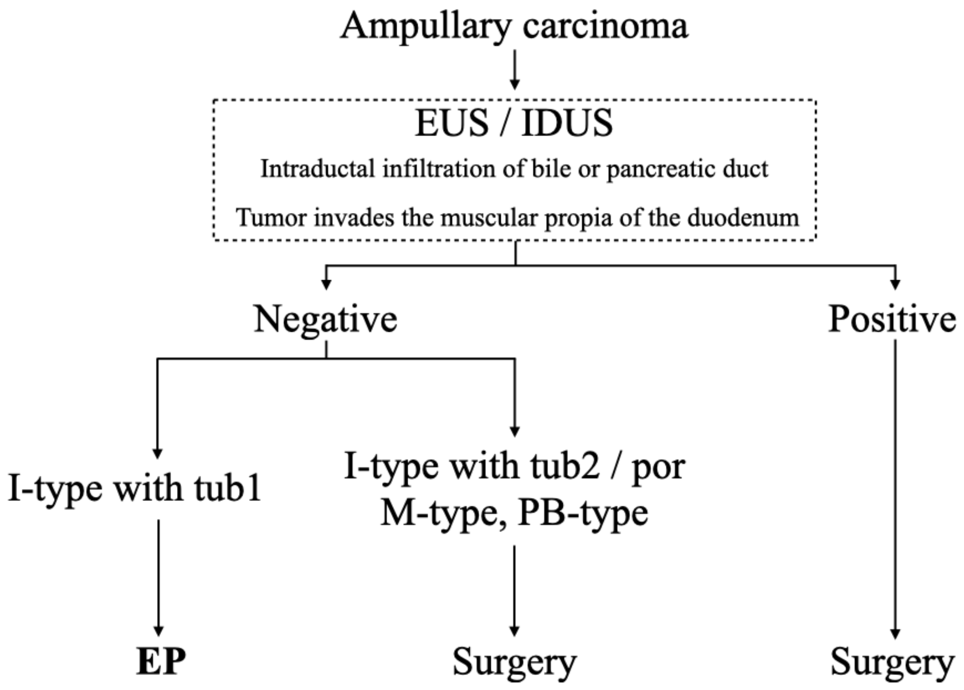

I-type: intestinal type, M-type: mixed type, PB, pancreatobiliary type; tub1, well differentiated tubular adenocarcinoma; tub2, moderately differentiated tubular adenocarcinoma; por, poorly differentiated adenocarcinoma; EUS, endoscopic ultrasonography; IDUS, intraductal ultrasonography; EP, endoscopic papillectomy

Supplementary Figure S6. Overall stage based on TNM clinical classification

Stage 0	Tis	N0	M0
Stage IA	T1a	N0	M0
Stage IB	T1b, T2	N0	M0
Stage IIA	T3a	N0	M0
Stage IIB	T3b	N0	M0
Stage IIIA	T1a, T1b, T2, T3	N1	M0
Stage IIIB	Any T	N2	M0
	T4	Any N	M0
Stage IV	Any T	Any N	M1

Note: WHO classification

Supplementary Figure S7. T stage of the primary tumor based on the TNM clinical classification

Tis	Carcinoma in situ
T1a	Tumor limited to ampulla of Vater or sphincter of Oddi
T1b	Tumor invades beyond the sphincter of Oddi and/or into the duodenal submucosa
T2	Tumor invades the muscular propia of the duodenum
T3	Tumour invades pancreas or peripancreatic tissue
T3a	Tumour invades 0.5 cm or less into the pancreas
T3b	Tumour invades more than 0.5cm into the pancreas or extends into peripancreatic tissue or duodenal serosa but without beyond involvement of the celiac axis or the superior mesenteric artery
T4	Tumor with vascular involvement of the superior mesenteric artery or celiac axis, or common hepatic artery

Note: WHO classification
